# The analgesic effect of ultrasound-guided cervical erector spinae block in arthroscopic shoulder surgery: a randomized controlled clinical trial

**DOI:** 10.1186/s12871-024-02586-7

**Published:** 2024-06-03

**Authors:** Manhua Zhu, Ruifen Zhou, Lingzhi Wang, Qilu Ying

**Affiliations:** 1 Department of Anesthesiology, The Affiliated LiHuiLi Hospital of Ningbo University, No.57 Xingning Road, Ningbo, Zhejiang 315040 China; 2 Health Science Center, Ningbo University, No.818 Fenghua Road, Ningbo, Zhejiang 315211 China

**Keywords:** Erector spinae plane block, Arthroscopic shoulder surgery, Pain, Postoperative analgesia

## Abstract

**Background:**

Erector spinae plane block (ESPB) is a novel fascial plane block technique that can provide effective perioperative analgesia for thoracic, abdominal and lumbar surgeries. However, the effect of cervical ESPB on postoperative analgesia after arthroscopic shoulder surgery is unknown. The aim of this study is to investigate the analgesic effect and safety of ultrasound-guided cervical ESPB in arthroscopic shoulder surgery.

**Methods:**

Seventy patients undergoing arthroscopy shoulder surgery were randomly assigned to one of two groups: ESPB group (*n* = 35) or control group (*n* = 35). Patients in the ESPB group received an ultrasound-guided ESPB at the C7 level with 30 mL of 0.25% ropivacaine 30 min before induction of general anesthesia, whereas patients in the control group received no block. The primary outcome measures were the static visual analogue scale (VAS) pain scores at 4, 12, and 24 h after surgery. Secondary outcomes included heart rate (HR) and mean arterial pressure (MAP) before anesthesia (t1), 5 min after anesthesia (t2), 10 min after skin incision (t3), and 10 min after extubation (t4); intraoperative remifentanil consumption; the Bruggrmann comfort scale (BCS) score, quality of recovery-15 (QoR-15) scale score and the number of patients who required rescue analgesia 24 h after surgery; and adverse events.

**Results:**

The static VAS scores at 4, 12 and 24 h after surgery were significantly lower in the ESPB group than those in the control group (2.17 ± 0.71 vs. 3.14 ± 1.19, 1.77 ± 0.77 vs. 2.63 ± 0.84, 0.74 ± 0.66 vs. 1.14 ± 0.88, all *P* < 0.05). There were no significant differences in HR or MAP at any time point during the perioperative period between the two groups (all *P* > 0.05). The intraoperative consumption of remifentanil was significantly less in the ESPB group compared to the control group (*P* < 0.05). The scores of BCS and QoR-15 scale were higher in the ESPB group 24 h after surgery than those in the control group (*P* < 0.05). Compared to the control group, fewer patients in the ESPB group required rescue analgesia 24 h after surgery (*P* < 0.05). No serious complications occurred in either group.

**Conclusions:**

Ultrasound-guided cervical ESPB can provide effective postoperative analgesia following arthroscopic shoulder surgery, resulting in a better postoperative recovery with fewer complications.

**Trial registration:**

Chictr.org.cn identifier ChiCTR2300070731 (Date of registry: 21/04/2023, prospectively registered).

## Introduction

Arthroscopy shoulder surgery has emerged as a common surgical procedure for treating shoulder diseases because of its minimally invasive and rapid post-operative recovery [1]. However, postoperative pain caused by intraoperative pressure irrigation and tissue swelling should not be underestimated. Furthermore, in order to reduce intraoperative bleeding and maintain a clear surgical field of view, intraoperative controlled hypotension is necessary [2]. Adequate perioperative analgesia can not only meet the needs of intraoperative controlled hypotension, but also improve postoperative early mobilization, accelerate functional exercise and rehabilitation, and shorten hospitalization stays [3]. Several analgesic methods and techniques have been employed for perioperative pain management in arthroscopy shoulder surgery, including patient-controlled intravenous analgesia (PCIA), interscalene brachial plexus and local infiltration anesthesia [4]. These analgesic methods have their own advantages, but they also carry the risk of inadequate analgesia, and can cause some serious complications, such as phrenic nerve block, dyspnea, central neuraxial nerve injury, and Horner’s syndrome, limiting their use in certain populations [5, 6]. Therefore, it is urgent to explore a more effective and safe analgesic method following arthroscopy shoulder surgery.

Since the initial report by Forero et al. in 2016 [7], erector spinae plane block (ESPB) has emerged as a novel fascial plane block technique. It is commonly used for postoperative analgesia in thoracic, abdominal, and spinal surgeries [8–11]. The local anesthetic (LA) is injected into the fascial plane between the erector spinae muscle and the transverse processes. LA spreads over 3–6 vertebral levels in the potential space to block the ventral and dorsal branches of the spinal nerve, and some of the liquid may spread to the paravertebral space, thereby producing analgesic effects [11].

Recently, a study conducted on cadavers found that cervical (C6, C7) erector spinae plane injections consistently stained the cervical 5–7 nerve roots [12], theoretically implying that the cervical ESPB has the potential to provide postoperative analgesia for shoulder surgery. However, no randomized controlled clinical trial has examined its clinical efficacy in arthroscopic shoulder surgery. The aim of this study is to investigate the feasibility, safety, and analgesic effect of ultrasound-guided cervical ESPB in patients undergoing arthroscopic shoulder surgery.

## Methods

This randomized prospective double-blind clinical trial was approved by the Affiliated LiHuiLi Hospital of Ningbo University Ethics Committee (identifier: KY2022PJ214). The study was registered on www.chictr.org.cn (ChiCTR2300070731). Written informed consent was provided by all subjects before participation in the study.

Patients undergoing arthroscopic shoulder surgery under general anesthesia were recruited between May 2023 and December 2023 in the Affiliated LiHuiLi Hospital of Ningbo University. Inclusion criteria: aged between 18 and 70 years; body mass index (BMI) between 18 and 32 kg/m^2^, American Society of Anesthesiologists Classification (ASA) Grade I to II; and scheduled to undergo arthroscopic shoulder surgery. Exclusion criteria: severe cardiopulmonary disease, allergy to LA, infection at the puncture site, bleeding diathesis, language communication disorder, cognitive impairment, and refusal to participate.

### Randomization and blinding

All subjects were randomly assigned into two groups using a computer-generated random number table 1 day before surgery: the ESPB group and the control group. A nerve block specialist with extensive experience performed the ESPB. The specialist was not involved in anesthesia management. Patients, surgeons, anesthesiologists, nurses, postoperative evaluators and statistical analysts were all blinded to the study.

### Ultrasound-guided ESPB

**Fig. 1:**
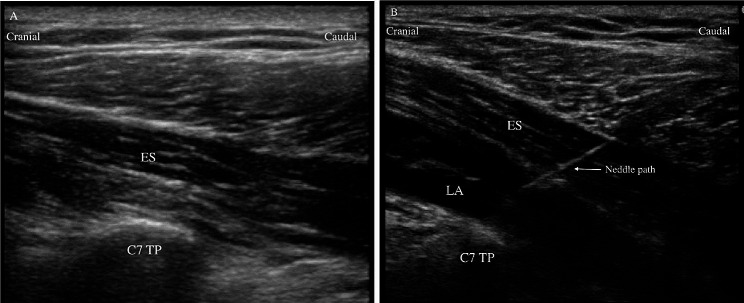
Image of ultrasound-guided C7 ESPB. (A) pre block. (B) post block. LA spreads between the C7 spinous process and the erector spinae muscle. ES, erector spinae muscle, LA, local anesthetic, C7 TP, C7 transverse process, and the arrow indicates the path of the needle

All patients underwent standard monitoring procedure which included an electrocardiogram (ECG), pulse oxygen saturation(SPO_2_), non-invasive arterial blood pressure (NIBP), and bispectral index (BIS) were performed in the operating room. A peripheral intravenous cannula pathway was established. Patients in the ESPB group underwent an ultrasound scan in the lateral decubitus position with the surgical side up, 30 min before general anesthesia induction. A linear ultrasound transducer (6–13 MHz, Edge, Sonosite, Seattle, USA) was positioned in a longitudinal sagittal plane at the level of C7-T1, 3–4 cm lateral to the posterior midline, to identify the 1st rib and the T1 transverse process. Then the transducer was moved to the cephalic side to identify the erector spinae muscle and the C7 transverse process. After disinfection and subcutaneous infiltration with 2% lidocaine (1 mL), a 21G×100 mm nerve block needle (Pajunk, Geisingen, Germany) was inserted in a caudal-to-cranial direction until its tip reached the interfascial plane between the erector spinae muscle and C7 transverse process using the in-plane technique (Fig. 1). After confirming the location of the needle tip with hydrodissection, a total of 30 mL of 0.25% ropivacaine (Naropin, AstraZeneca AB Company, Södertälje, Sweden) was slowly administered. In contrast, patients in the control group only received an ultrasound scan of the erector spinae muscles at the C7 level, as well as subcutaneous infiltration with normal saline (1 mL) but no nerve block needle was inserted. All patients had a motor block test and dermatomal evaluation of sensory block with a cold test 20 min after ESPB. Motor and sensory block tests were performed by a nurse anesthetist who was not involved in the study.

### General anesthesia and analgesia protocol

Both groups underwent standardized general anesthesia. To induce general anesthesia, intravenous 0.04 mg/kg midazolam, 2 mg/kg propofol, 0.3–0.4 µg/kg sufentanil, and 0.6 mg/kg rocuronium was administered. Endotracheal intubation was performed after achieving adequate muscle relaxation, followed by mechanical ventilation. To maintain general anesthesia, a continuous intravenous infusion of propofol 0.1–0.15 mg/kg/min and remifentanil 0.1–0.2 µg/kg/min was used to keep the BIS at 40–60. Remifentanil dosage was adjusted to maintain hemodynamic parameters [heart rate (HR) and mean blood pressure (MAP)] within 20% of the baseline.

Both groups were treated with the same postoperative multimodal pain management protocol. 0.15ug/kg sufentanil was administered intravenously 10 min before the end of the surgery. Following surgery, patients were given 40 mg of parecoxib sodium intravenously every 12 h, and treated with acupoint application and needle embedding. If the static visual analogue scale (VAS) score was greater than 4, tramadol 100 mg was given intramuscularly for remedial analgesia.

### Outcomes

The primary outcome measure was the static VAS pain score (0–10: 0, painless,10, excruciating pain) at 4, 12, and 24 h after surgery.

Secondary outcomes included HR and MAP before anesthesia (t1), 5 min after anesthesia (t2), 10 min after skin incision (t3), and 10 min after extubation (t4); intraoperative remifentanil consumption; the Bruggrmann comfort scale (BCS) score, quality of recovery-15 (QoR-15) scale score, number of patients requiring rescue analgesia and adverse events 24 h after surgery [LA systemic toxicity (LAST), respiratory depression, postoperative nausea and vomiting (PONV), dizzy and pruritus].

The BCS (0–4; 0, persistent pain; 1, severe pain when breathing deeply or coughing; 2, mild pain when breathing deeply or coughing; 3, painless when breathing deeply; and 4 painless when coughing) was used to assess patients’ comfort level.

The QoR-15 scale was used to evaluated the overall quality of postoperative recovery. It includes 15 items to measure five dimensions: physical comfort, emotional state, physical independence, psychological support and pain. The scores range from 0 to 150, with higher scores indicating better recovery [13].

### Statistical analysis

A preliminary study of 10 patients in each group found that the mean and standard deviation (SD) of VAS score was 2.1 (1.6) in the ESPB group and 2.9 (1.1) in the control group at 12 h after surgery. According to the findings, a sample of 28 participants was required in each group to achieve a statistical power of 0.8 and a two-sided (two-tailed) type I error of 0.05. Therefore, we planned to recruit 35 subjects in each group to account for possible dropouts. The sample size was estimated with IBM SPSS Sample Power version 3.0 (IBM Corp., Armonk, New York, USA).

Normality was evaluated with the Kolmogorov-Smirnov test. Normally distributed continuous variables were analyzed with the two-sample *t*-test and reported as mean ± SD (VAS scores, HR, MAP, the consumption of remifentanil, BCS scores, and QoR-15 scale score). A repeated-measures analysis of variance was used to compare groups at different time points (VAS scores, HR and MAP). Categorical variables were analyzed with the chi-square (*χ2*) test or Fisher’s exact test and reported as frequency (percentage) (rescue analgesia requirement, the incidence of PONV, dizzy and pruritus). We calculated a 95% confidence interval (CI) for differences in means (for continuous variables) or relative risk (RR) (for categorical variables). A *P*-value of < 0.05 was considered statistically significant. SPSS V.25.0 (IBM Corp., Armonk, New York, USA) was used to perform statistical analyses.

## Results

**Fig. 2:**
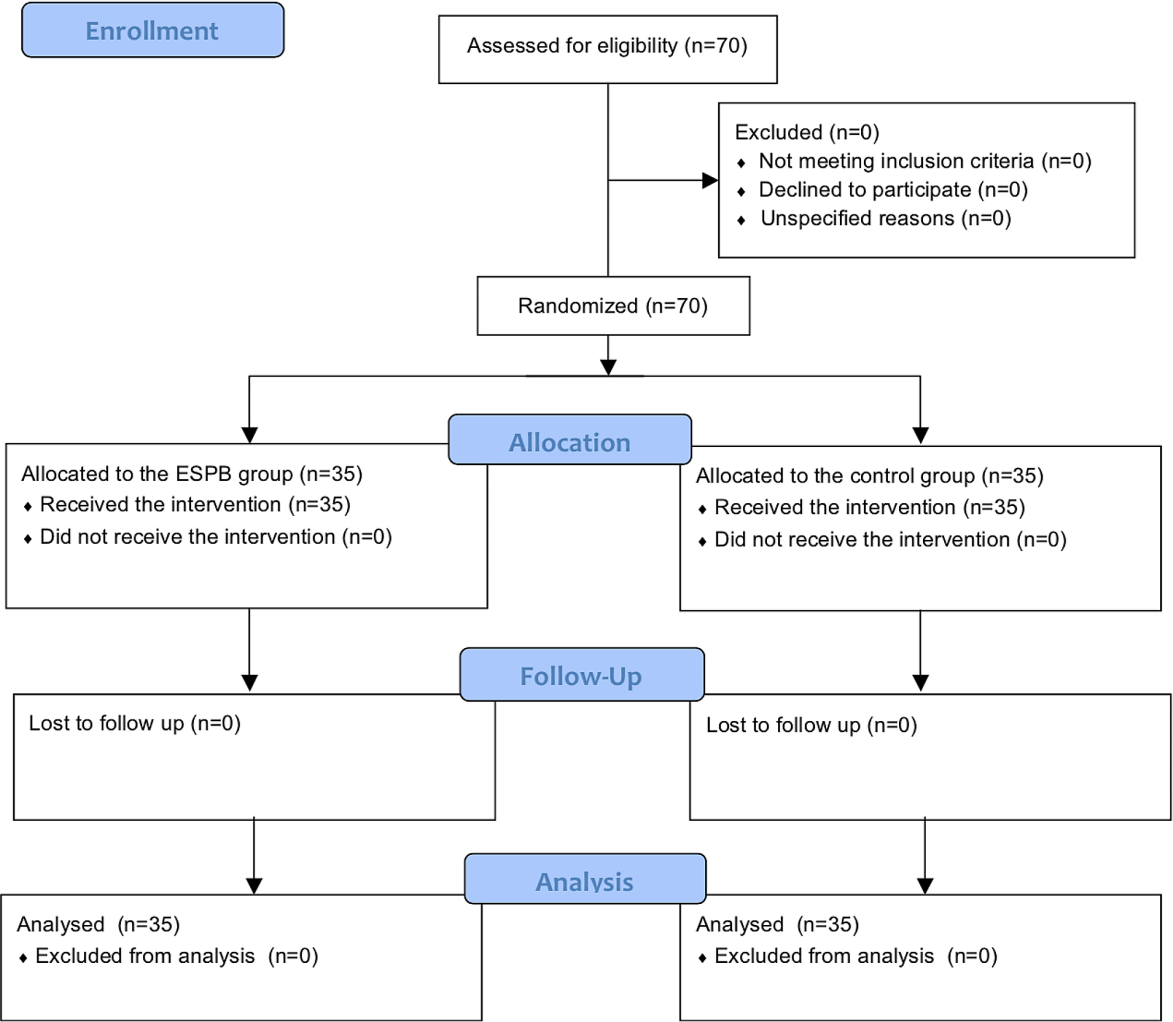
CONSORT diagram. ESPB, erector spinae plane block

A total of 70 subjects were screened for eligibility and randomly assigned to either the ESPB group or the control group. All subjects completed the study and were analyzed for the outcomes. The Consolidated Standards of Reporting Trials flow diagram for this study is depicted in Fig. 2. The baseline and demographic characteristics were comparable between the two groups (Table 1).

**Table 1:** Baseline and demographic characteristics

	ESPB group (*n* = 35)	control group(*n* = 35)	*p*
Age (years)	56.1 ± 8.4	54.3 ± 9.2	0.311
Gender			0.322
Male	11 (31.4)	15 (42.9)	
Female	24 (68.6)	20 (57.1)	
BMI (kg/m^2^)	23.52 ± 2.14	24.48 ± 2.73	0.104
ASA			> 0.999
I	7 (20.0)	7 (20.0)	
II	28 (80.0)	28 (80.0)	
Surgical side			0.473
Left	18 (51.4)	15 (42.9)	
Right	17 (48.6)	20 (57.1)	
Surgical time (min)	64.67 ± 27.8	71.5 ± 44.4	0.442

The data are represented as the mean ± SD or number (percentage)

ESPB, erector spinae plane block; BMI, body mass index; ASA, American Society of Anesthesiologists; n, number; SD, standard deviation

Continuous data were compared by Student’s t-test; categorical data were compared using the chi-square (χ^2^) test

### Primary outcome

The effects of group, time and the interaction on the static VAS scores between the two groups were all significant (*P* < 0.05, Table 2). Through multiple comparisons, the static VAS scores at 4, 12, and 24 h after surgery were significantly lower in the ESPB group when compared with the control group (*P* < 0.05, Table 2). The static VAS scores in both groups at 4 h were significantly higher than that at 12 h and 24 h after surgery (*p* < 0.05), and the static VAS score at 12 h after surgery was significantly higher than that at 24 h after surgery (*P* < 0.05).

**Table 2:** The postoperative static VAS pain scores

static VAS scores	ESPB group (*n* = 35)	control group (*n* = 35)	F	*p*	Mean Difference (95%CI)
4 h after surgery	2.17 ± 0.71^#^	3.14 ± 1.19		0.000	-0.97 (-1.44, -0.50)
12 h after surgery	1.77 ± 0.77^#^	2.63 ± 0.84		0.000	-0.86 (-1.24, -0.47)
24 h after surgery	0.74 ± 0.66^#^	1.14 ± 0.88		0.035	-0.40 (-0.77, -0.03)
Intergroup effect			16.682	0.000	
Time effect			231.001	0.000	
Interaction effect			6.700	0.002	

The data are represented as the mean ± SD, mean difference (95%CI)

ESPB, erector spinae plane block; CI, confidence interval; VAS, visual analogue scale; n, number; SD, standard deviation

Compared with the control group, ^#^*P* < 0.05. Data were compared by repeated-measures analysis of variance

### Secondary outcomes

The time had statistically significant effect on HR and MAP (*P* < 0.05, Table 3). However, the intergroup effect and the interaction effect were not significant (*P* > 0.05, Table 3). Through multiple comparisons, there were no differences in HR or MAP at any time during the perioperative period between the two groups (*P* > 0.05, Table 3). The HR between the two groups at t1 was significantly higher than at t2 and t3 (*p* < 0.05), and the HR at t4 was significantly higher than at t3 (*P* < 0.05). The MAP between the two groups at t1 was significantly higher than at t2, t3 and t4 (*p* < 0.05), and the MAP at t4 was significantly higher than at t2 and t3 (*P* < 0.05).

**Table 3:** Perioperative HR and MAP

	ESPB group (*n* = 35)	control group (*n* = 35)	F	*p*	Mean Difference (95%CI)
HR (bpm)					
t1	71.69 ± 10.33	74.14 ± 13.19		0.389	-2.46 (-8.11, 3.20)
t2	62.80 ± 7.89	66.17 ± 12.69		0.186	-3.37 (-8.41, 1.67)
t3	58.49 ± 9.31	62.91 ± 11.71		0.084	-4.43 (-9.48, 0.62)
t4	68.37 ± 10.23	70.11 ± 14.14		0.083	-5.37 (-11.46, 0.72)
intergroup effect			4.030	0.051	
time effect			21.265	0.000	
interaction effect			0.321	0.781	
MAP (mmHg)					
t1	111.43 ± 14.86	108.94 ± 12.42		0.450	2.49 (-4.05, 9.02)
t2	89.71 ± 16.61	86.20 ± 14.09		0.343	3.51 (-3.83, 10.86)
t3	88.80 ± 12.42	85.26 ± 13.81		0.263	3.54 (-2.72, 9.81)
t4	99.29 ± 15.54	98.77 ± 15.91		0.892	0.51 (-6.99, 8.02)
intergroup effect			1.328	0.253	
time effect			39.098	0.000	
interaction effect			0.165	0.919	

The data are represented as the mean ± SD, mean difference (95%CI)

bpm, beat per minute; ESPB, erector spinae plane block; CI, confidence interval; HR, heart rate; MAP, mean arterial pressure, n, number; SD, standard deviation

Data were compared using repeated-measures analysis of variance

The intraoperative consumption of remifentanil was significantly lower in the ESPB group as compared with the control group (*P* < 0.05). The scores of BCS and QoR-15 scale were higher in the ESPB group 24 h after surgery than those in the control group (*P* < 0.05). Compared to the control group, fewer patients in the ESPB group required rescue analgesia 24 h after surgery (*P* < 0.05, Table 4).

No serious complications, such as respiratory depression or LAST, occurred in either group. The incidence of postoperative complications including PONV, dizziness and pruritus was similar between the two groups(*P*>0.05, Table 5).

**Fig. 3:**
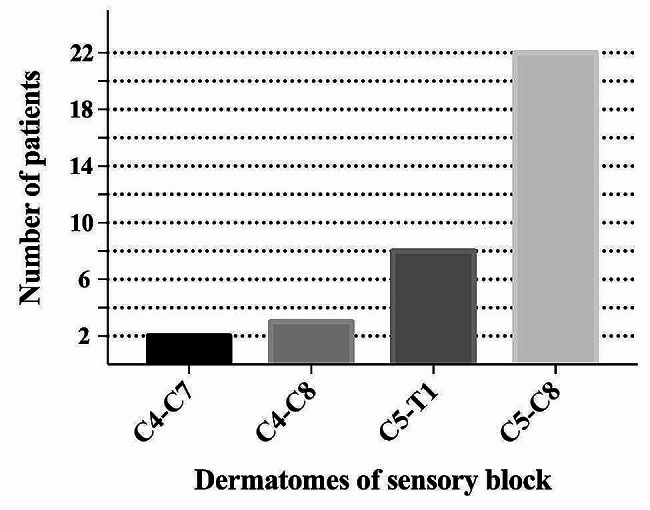
Affected dermatomes in patients who received ESPB

No patients in the ESPB group experienced muscle weakness in the upper limb. We observed a range of sensory blocks in patients who received ESPB, the affected dermatomes were located between C4 and T1, primarily between C5 and C8 (Fig. 3). Seven patients had loss of sensation up to the midclavicular line, twenty patients had loss of sensation beyond the anterior axillary line, and eight patients contained to the posterior trunk in the ESPB group.

## Discussion

The results showed that ESPB at the C7 level can provide effective postoperative analgesia following arthroscopic shoulder surgery, lowering pain scores, improving comfort scale and the quality of postoperative recovery, and reducing the need for postoperative rescue analgesia with fewer complications.

Arthroscopic shoulder surgery may have possible advantages over traditional shoulder open surgery because it involves a small incision, less deltoid injury, and faster postoperative recovery. However, in terms of postoperative pain and the need for analgesics, the two surgical techniques may be comparable [14]. Severe postoperative pain can interfere with early postoperative functional rehabilitation training.

As a regional interfascial plane block, ESPB has shown promise as a part of multimodal analgesia for a variety of surgeries with good effect. ESPB was proposed for treating chronic shoulder pain in 2018 [15]. A recent cadaver study found that the brachial plexus roots of C5, C6 and C7 were all deeply stained when the dye was injected at the erector spinae plane at C6 or C7 level, whereas C4, C8, and T1 roots were only stained in a few samples [12]. The neuronal innervation of the shoulder is provided by branches of the cervical and the brachial plexuses [16]. The main sensory nerves of the shoulder are the supraclavicular (C3–C4) and axillary (C5–C6) nerves [16]. In this study, we discovered that ESPB at the C7 level can block the sensory nerves from C4–T1, which was consistent with the findings of the preceding cadaveric study, implying that C7 level ESPB may target the neuronal innervation of the shoulder and provide effective analgesia after arthroscopic shoulder surgery. Furthermore, the results showed that the C7 ESPB led to a sensory, but not motor block, which could be attributed to low LA concentration. Our findings indicating that ESPB with 0.25% ropivacaine could block sensory nerves but maintain motor capacity.

It has been reported that the analgesic effect of ESPB may be related to the amount of LA volume [17]. Large volume ESPB may allow for the effective spread of LA in the fascial plane. Ciftci et al. [18] reported that by injecting 30 mL of LA at the T2 level can increase the skin sensory block range to C3–T3. Therefore, we chose a volume of 30 mL for cervical ESPB in this study.

Our findings showed that the static VAS scores were significantly lower and fewer patients required rescue analgesia in the ESPB group than in the control group 24 h after surgery, which was in consistent with the study by Ciftci et al. [18], who reported that high thoracic ESPB significantly reduces VAS score and the need for rescue analgesia after arthroscopic shoulder surgery. In addition, Hamadnalla et al. [19] performed continuous ESPB at the C7 level for shoulder disarticulation surgery in one patient, discovered a loss of cold sensation from C4–T2 and reported successful pain management. Tsui et al. [20] placed a cervical ESPB catheter using a thoracic approach for forequarter amputation in an old female, and they reported complete relief of shoulder pain and high-quality postoperative pain control. Similar to the findings above, our study found C7 ESPB to be an effective option for early postoperative analgesia following arthroscopic shoulder surgery.

The intraoperative remifentanil consumption was significantly less and the BCS scores were higher in the ESPB group than in the control group. The results are similar to those of Hu et al. [21], who reported that ESPB applied for a cesarean section can significantly reduce the required dose of remifentanil while increasing postoperative BCS scores. This suggests that ESPB can reduce intraoperative opioid consumption while improving patient comfort. In this study, the quality of recovery was evaluated using the QoR-15 scale score. We found that patients who received an ESPB for arthroscopic shoulder surgery had a significantly higher quality of recovery, which could be attributed to the reduced opioid use and lower pain scores in the ESPB group.

**Table 4:** Remifentanil consumption, BCS, QoR-15 scale score, and rescue analgesia requirements

	ESPB group (*n* = 35)	control group (*n* = 35)	Mean Difference (95%CI) or RR (95%CI)		*P*
Remifentanil (mg)	0.59 ± 0.21^#^	0.76 ± 0.33	-0.17 (-0.30, -0.35)	0.014	
BCS score	1.71 ± 0.93^#^	1.26 ± 0.92	0.46 (0.02, 0.90)	0.046	
QoR-15 scale score	137.37 ± 6.49^#^	131.94 ± 9.66	5.43 (1.49, 9.36)	0.007	
rescue analgesia requirement	2(5.7)^#^	9(22.9)	0.22 (1.03–1.57)	0.045	

The data are represented as the mean ± SD, mean difference (95%CI) or RR (95%CI)

ESPB, erector spinae plane block; CI, confidence interval; RR, relative risk; BCS, Bruggrmann comfort scale; QoR-15, quality of recovery-15 scale; n, number; SD, standard deviation

Compared with the control group, ^#^*P* < 0.05. Data were compared using Student’s t-test, while categorical data were compared using Fisher’s exact test

**Table 5:** Incidence of adverse events

	ESPB group (*n* = 35)	control group (*n* = 35)	RR (95%CI)	*P*
LAST	0	0		
respiratory depression	0	0		
PONV	1 (2.9)	6 (17.1)	0.17 (0.02, 1.31)	0.106
dizziness	1 (2.9)	3 (8.6)	0.39 (0.04, 3.51)	0.618
pruritus	3 (8.6)	7(20)	0.54 (0.12, 1.52)	0.306

The data are represented as a number (percentage), RR (95%CI)

ESPB, erector spinae plane block; RR, relative risk; CI, confidence interval; LAST, local anesthetic systemic toxicity; PONV, postoperative nausea and vomiting; n, number

Data were compared using Fisher’s exact test

No severe ESPB-related complications were observed, indicating that ESPB at the C7 level is relatively safe, possibly due to the needle’s target being the transverse process and injection being well away from the spinal canal and blood vessels. In this study, we first investigated cervical ESPB after arthroscopic shoulder surgery, and recommend the use of cervical ESPB in arthroscopic shoulder surgery.

This study also has some limitations. First, due to the study’s short follow-up period, the long-term effects of cervical ESPB on patient outcomes warrant further investigation. Second, we only used single-injection blocks in our study. Further research is needed to confirm the effectiveness of continuous cervical ESPB. Third, this trial was conducted in a single center with a small sample size, so the findings should be further confirmed in multicenter trials with large sample sizes.

## Conclusions

ESPB at the C7 level provides effective postoperative analgesia for patients undergoing arthroscopic shoulder surgery. C7 ESPB has been shown to reduce postoperative pain scores, increase patient comfort, and improve recovery quality. Further research is needed to determine the optimal technique and LA required for an effective and safe cervical ESPB.

## Data Availability

The datasets used during the current study are available from the corresponding author on reasonable request.

## Abbreviations

ASA: America Society of Anesthesiologist
BCS: Bruggrmann comfort scale
BIS: Bispectral index
BMI: Body mass index
CI: Confidence interval
ECG: Electrocardiogram
ES: Erector spinae muscle
ESPB: Erector spinae plane block
HR: Heart rate
LAST: Local anesthetic systemic toxicity
LA: Local anesthetic
MAP: Mean arterial pressure
NIBP: Non-invasive arterial blood pressure
PCIA: Patient-controlled intravenous analgesia
PONV: Postoperative nausea and vomiting
QoR: 15-quality of recovery-15
RR: Relative risk
SD: Standard deviation
SPO2: Pulse oxygen saturation
TP: Transverse process
VAS: Visual analogue scale
